# Mitochondrial metabolism and cancer

**DOI:** 10.1038/cr.2017.155

**Published:** 2017-12-08

**Authors:** Paolo Ettore Porporato, Nicoletta Filigheddu, José Manuel Bravo-San Pedro, Guido Kroemer, Lorenzo Galluzzi

**Affiliations:** 1 Department of Molecular Biotechnology and Health Sciences, Molecular Biotechnology Center, 10124 Torino, Italy; 2 Department of Translational Medicine, University of Piemonte Orientale, 28100 Novara, Italy; 3 Université Paris Descartes/Paris V, Sorbonne Paris Cité, 75006 Paris, France; 4 Université Pierre et Marie Curie/Paris VI, 75006 Paris, France; 5 Equipe 11 labellisée par la Ligue contre le Cancer, Centre de Recherche des Cordeliers, 75006 Paris, France; 6 INSERM, U1138, 75006 Paris, France; 7 Metabolomics and Cell Biology Platforms, Gustave Roussy Comprehensive Cancer Institute, 94805 Villejuif, France; 8 Pôle de Biologie, Hopitâl Européen George Pompidou, AP-HP, 75015 Paris, France; 9 Department of Women's and Children's Health, Karolinska University Hospital, 17176 Stockholm, Sweden; 10 Department of Radiation Oncology, Weill Cornell Medical College, New York, NY 10065, USA; 11 Sandra and Edward Meyer Cancer Center, New York, NY 10065, USA

**Keywords:** autophagy, danger signaling, immunometabolism, oncometabolites, oxidative phosphorylation, mitophagy

## Abstract

Glycolysis has long been considered as the major metabolic process for energy production and anabolic growth in cancer cells. Although such a view has been instrumental for the development of powerful imaging tools that are still used in the clinics, it is now clear that mitochondria play a key role in oncogenesis. Besides exerting central bioenergetic functions, mitochondria provide indeed building blocks for tumor anabolism, control redox and calcium homeostasis, participate in transcriptional regulation, and govern cell death. Thus, mitochondria constitute promising targets for the development of novel anticancer agents. However, tumors arise, progress, and respond to therapy in the context of an intimate crosstalk with the host immune system, and many immunological functions rely on intact mitochondrial metabolism. Here, we review the cancer cell-intrinsic and cell-extrinsic mechanisms through which mitochondria influence all steps of oncogenesis, with a focus on the therapeutic potential of targeting mitochondrial metabolism for cancer therapy.

## Introduction

With the advent of the twenty-first century, two major misconceptions about cancer have eventually been eradicated: (1) the notion that cancer is a purely cell-intrinsic disorder that stems from epigenetic or genetic alterations^1,2^; and (2) the view that malignant cells satisfy their bioenergetic and anabolic needs mostly (if not only) via aerobic glycolysis^3,4^. Thus, it is now widely accepted that tumors form, develop and respond to therapy in the context of a complex, bidirectional interaction with the host immune system^5,6^. Similarly, the fundamental influence of mitochondrial metabolism on all steps of oncogenesis, i.e., malignant transformation, tumor progression and response to treatment, has eventually been given proper recognition^7,8^.

Interestingly, the roots of these long-standing misconceptions reside in two notions that *de facto* revolutionized (in the positive sense of the term) modern medicine: (1) the “self/non-self” dichotomy, as originally theorized by the Australian virologist Sir Frank Macfarlane Burnet (1899-1985) in 1949, proposing that the immune system can only recognize foreign entities^9,10^; and (2) the so-called “Warburg effect”, referring to the elevated uptake of glucose that characterizes a majority of cancers, first described by the German physiologist Otto Heinrich Warburg (1883-1970) in 1927^11,12^. The self/non-self theory generated a robust theoretical framework that turned out to be essential for our current understanding of immune responses against invading pathogens^9^, while the Warburg effect provided the rationale for the development of an imaging tool that has been (and still is) extensively used in the clinics for the detection and monitoring of neoplasms, 2-[^18^F]fluoro-2-deoxy-𝒟-glucose (^18^F-FDG) positron emission tomography (PET)^11^.

Despite limited experimental support^12,13^, Warburg himself suggested that the ability of malignant cells to maintain elevated glycolytic rates in spite of normal oxygen tension would derive from primary mitochondrial defects^14^, an incorrect assumption that *de facto* relegated mitochondria to a role of mere bystanders of the oncogenic process for decades. Renovated interest in the role of mitochondria in cancer came in the mid-1990s with the demonstration that mitochondrial outer membrane permeabilization (MOMP) constitutes a decisive step in the execution of regulated cell death (RCD)^15,16,17,18^. This discovery drove an intense wave of investigation that — only a few years later — culminated with the recognition that most (if not all) cancer cells display an accrued resistance to RCD often owing to alterations in the mitochondrial control of the process^19^. As a consequence, considerable efforts were focused on the development of molecules that would target mitochondria as a strategy for chemo- or radio-sensitization^20^, and some of these agents are nowadays used in the clinics (e.g., venetoclax, which is currently approved for use in patients with chronic lymphocytic leukemia)^21^. Alongside, mitochondria attracted renovated attention from a metabolic perspective, in particular as it became clear that: (1) some mitochondrial metabolites are sufficient to drive oncogenesis^22^, and (2) some mitochondrial circuitries can adapt to serve bioenergetic or anabolic functions, hence endowing malignant cells with considerable metabolic plasticity^23,24^. Thus, mitochondrial metabolism now stands out as a promising target for the development of novel antineoplastic agents, and several venues are currently being explored in this sense^25,26^.

One of the main problems with targeting mitochondria as a strategy to kill malignant cells or sensitize them to treatment is that multiple immune effector cells, and in particular CD8^+^ cytotoxic T lymphocytes (CTLs, which are involved in the efficacy of many — if not all — therapies), display remarkable metabolic similarities to cancer cells^26,27^. This calls for the development of refined therapeutic approaches whereby malignant cells are selectively targeted while immune cells are spared from (or rendered insensitive to) the detrimental effects of treatment. Here, we critically review the cancer cell-intrinsic and cell-extrinsic mechanisms whereby mitochondria influence malignant transformation, tumor progression and response to treatment, as we discuss the potential of targeting mitochondrial metabolism for cancer therapy.

## Mitochondrial metabolism in malignant transformation

The term “malignant transformation” generally refers to the conversion of a normal cell into a neoplastic precursor that — in the context of failing immunosurveillance — acquires additional alterations enabling unrestricted proliferative potential, dissemination, and formation of distant macrometastases (cumulatively referred to as “tumor progression”)^28^. Importantly, only carcinogen- and transgene-driven models of oncogenesis can recapitulate (albeit with several limitations) malignant transformation. Conversely, widely employed transplantable models including transformed cells of human or rodent origin *de facto* recapitulate late tumor progression only (as they were derived from primary or metastatic lesions that evaded immunosurveillance)^29^. Mitochondria may contribute to malignant transformation by at least three major mechanisms: (1) mitochondrial reactive oxygen species (ROS) favor the accumulation of potentially oncogenic DNA defects and the activation of potentially oncogenic signaling pathways^30^; (2) the abnormal accumulation of specific mitochondrial metabolites, including fumarate, succinate, and 2-hydroxyglutarate (2-HG), has prominent transforming effects (at least in some models)^31^; (3) functional deficits in MOMP or mitochondrial permeability transition (MPT) are generally required for the survival of neo-formed malignant precursors, which would otherwise succumb to RCD^32,33^.

ROS are established genotoxins^30^, and their requirement for malignant transformation is well exemplified by the fact that *Trp53^−/−^* mice maintained in relatively hypoxic conditions (10% O_2_) exhibit a considerable survival advantage secondary to markedly reduced level of tumorigenesis as compared to *Trp53^−/−^* mice maintained in standard atmospheric conditions (21% O_2_)^34^. Along similar lines, hypoxia inhibits spontaneous intestinal carcinogenesis in *Apc^Min/+^* mice as well as carcinogen-driven oncogenesis in wild-type BALB/c mice^34^. Moreover, mitochondrial DNA (mtDNA) mutations that mildly (but not severely) affect various components of the electron transport chain (ETC) as they promote ROS generation have been documented in a variety of tumors^8,35^. One of the major mechanisms that control mitochondrial fitness (and hence limit ROS production) is the autophagic removal of damaged mitochondria (commonly known as mitophagy)^36^. In line with this notion, the knockdown or knockout of genes that are essential for autophagy (such as *Atg5* or *Atg7*) can promote oncogenesis in specific contexts^37,38,39^. Moreover, Fanconi anemia (FA) genes — which are mutated or silenced in a large proportion of human tumors — have recently been shown to be involved in mitophagy^40^, suggesting that (at least part of) the oncosuppressive activity of FA proteins may stem from the proficient removal of damaged mitochondria overproducing ROS. Besides favoring mutagenesis, ROS trigger potentially oncogenic signal transduction cascades including mitogen-activated protein kinase (MAPK)^28^ and epidermal growth factor receptor (EGFR) signaling^41^.

Succinate dehydrogenase complex iron sulfur subunit B (SDHB), fumarate hydratase (FH), isocitrate dehydrogenase (NADP(+)) 1, cytosolic (IDH1) and isocitrate dehydrogenase (NADP(+)) 2, mitochondrial (IDH2) are affected by germline or somatic mutations in a variety of human tumors^31^. While SDHB and FH are generally hit by loss-of-function mutations, accompanied by the accumulation of fumarate and/or succinate, IDH1 and IDH2 frequently display gain-of-function mutations, leading to the synthesis of 2-HG^42^. Fumarate, succinate and 2-HG behave as *bona fide* oncometabolites, meaning that their accumulation is sufficient to drive malignant transformation (at least in some models)^42^. All these oncometabolites share the capacity to inhibit α-ketoglutarate (α-KG)-dependent enzymes that control gene expression at the epigenetic level, such as Jumonji domain (JMJ) histone lysine demethylases as well as ten-eleven translocation (TET) dioxygenases^43,44^, resulting in the expression of a potentially oncogenic transcriptional program associated with a block in terminal differentiation^42,45,46^. Moreover, 2-HG alters the α-KG-dependent prolyl oxidase activity of egl-9 family hypoxia inducible factor 1 (EGLN1, best known as PHD2) and EGLN2 (best known as PHD1), hence promoting transformation via a mechanism related to hypoxia inducible factor 1 alpha subunit (HIF1A) stabilization or destabilization^44,47^. Finally, fumarate can also induce a non-enzymatic post-translational protein modification known as “succination”, and succination of kelch like ECH-associated protein 1 (KEAP1) activates the oncogenic transcription factor nuclear factor, erythroid derived 2 (NFE2, best known as NRF2)^48^. Interestingly, the accumulation of succinate and fumarate does not always result from primary mitochondrial defects, but can also derive from signals dispatched from oncogenic proteins such as KRAS^49,50^. Along similar lines, loss of oncosuppressor genes such as *APC* appears to favor malignant transformation also by altering mitochondrial functions^51^.

Alterations in the susceptibility of mitochondria to undergo MOMP or MPT accompany a vast majority of human tumors, and they are required for malignant precursors to avoid oncogene-driven RCD^32,33^. Perhaps the most striking example of such alterations derives from the overexpression of BCL2 apoptosis regulator (BCL2), a multifunctional cytoprotective protein that localizes to the mitochondrial outer membrane^32^. Malignant transformation (as well as tumor progression, see below) in the hematopoietic system is often associated with the overexpression of BCL2 or other members of the BCL2 protein family, and this increases considerably the resistance of malignant precursors (as well as established cancer cells) to RCD, at least in part owing to an improved bioenergetic metabolism^52,53^. In a subset of follicular lymphoma patients, a chromosomal rearrangement involving *BCL2* (normally on chromosome 18) and the immunoglobulin heavy chain locus (normally on chromosome 14) — the so-called t(14;18) translocation^54^ — can be detected in a vast majority of blasts, suggesting that it constitutes a very early event in oncogenesis. Many oncogenes beyond *BCL2* (e.g., *MYC*, *KRAS*) drive malignant transformation as they increase the resistance of the mitochondrial pool to MOMP or MPT, in some cases via a mechanism that alters mitochondrial dynamics^55,56,57^. Besides triggering RCD, oncogene activation can also promote a permanent proliferative arrest known as cellular senescence, generally as a result of increased oxidative stress^58^. Cancer cells can evade such a response, as they activate pyruvate dehydrogenase kinase 1 (PDK1) or inhibit pyruvate dehydrogenase phosphatase catalytic subunit 2 (PDP2), resulting in limited pyruvate utilization by mitochondria and reduced ROS production^59^.

**Figure 1:**
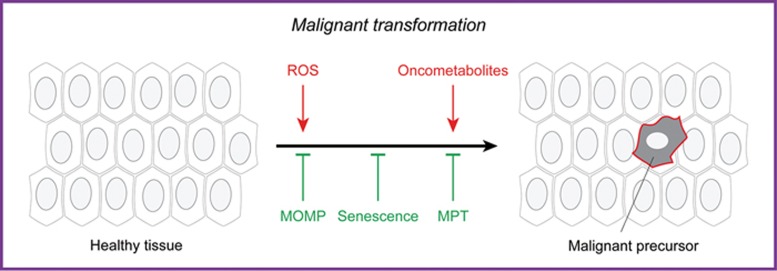
Mitochondrial metabolism in malignant transformation. Mitochondrial dysfunction can promote malignant transformation, i.e., the conversion of a healthy cell into a malignant precursor, as a consequence of (1) reactive oxygen species (ROS) overgeneration, which favors mutagenesis; (2) accumulation of succinate, fumarate or 2-hydroxyglutarate (all of which can operate as oncometabolites, at least in some settings); and/or (3) increased resistance to oncogene-driven mitochondrial outer membrane permeabilization (MOMP)- or mitochondrial permeability transition (MPT)-driven regulated cell death or cellular senescence.

Altogether, these observations exemplify the critical influence of mitochondria on malignant transformation (Figure 1).

## Mitochondrial metabolism in tumor progression

Mitochondria are the key for virtually all facets of tumor progression, not only as a major source of ATP, but also due to (1) their ability to provide building blocks for anabolism via anaplerosis, (2) their capacity to produce ROS, and (3) their central position in RCD signaling. In line with this notion, the ability of mtDNA-depleted (ρ^0^) cells to form tumors upon inoculation in immunocompatible hosts is compromised^60,61,62^, but can be recovered (at least in some settings) upon horizontal transfer of whole mitochondria from the host^60,63^. Along similar lines, severe defects in autophagy or mitophagy — resulting in fully compromised mitochondrial functions — have been associated with decreased tumor progression in multiple models of oncogenesis^39,64,65,66^.

### Proliferation

Although *in vitro*, under optimal growth conditions (which differ significantly from those encountered in the tumor microenvironment *in vivo*), cancer cells can obtain sufficient ATP from glycolysis, mitochondria are required for proliferation unless supraphysiological amounts of uridine and pyruvate are exogenously provided^67^ to compensate for pyrimidine and aspartate biosynthesis^68,69^. Progressing tumors display indeed an extensive and highly plastic metabolic rewiring. This involves not only increased uptake of glucose, a fraction of which is redirected to the pentose phosphate pathway (PPP) for nucleic acid synthesis and glutathione reduction^70^, but also the ability to process glutamine oxidatively (for energy production via the Krebs cycle and the ETC) or reductively (for fatty acid synthesis, cholesterol synthesis and the maintenance of oxidative homeostasis via NADPH production)^71,72,73,74^, the ability to flexibly use various other carbon sources including (but perhaps not limited to) acetate, lactate, serine and glycine as needed^75,76,77,78,79^, and the ability to interchangeably use glycolysis, oxidative phosphorylation (OXPHOS) and fatty acid oxidation as the source of energy in response to fluctuating microenvironmental conditions (such as local acidosis, which inhibits glycolysis)^80^.

The reversibility of many reactions of the tricarboxylic acid (TCA) cycle and the existence of multiple anaplerotic circuitries centered on mitochondria ensure such a metabolic adaptation^25,81^. One key TCA intermediate in this respect is citrate, because it resides at a crucial intersection between catabolic and anabolic metabolism, and hence operates as a major node of flexibility^82^. Besides fueling the oxidative mode of the TCA, citrate can also be converted into acetyl-CoA for export to the cytoplasm and nucleus^4,81^, where it can either be employed for fatty acid and cholesterol synthesis (to support the membrane need associated with intense proliferation) or used for acetylation reactions, which regulate transcription as well as cytoplasmic processes including autophagy^36,83,84^. In line with this notion, the enzyme that converts citrate into acetyl-CoA, i.e., ATP citrate lyase (ACLY), is required for cancer cells to proliferate at optimal rates^85^, but not for normal cells to do so (owing to a glucose-to-acetate metabolic switch)^86^. Reductive glutamine metabolism is the major source of citrate in the presence of mitochondrial defects, as well as under hypoxic conditions (as a function of the α-KG/citrate ratio)^23,73,87^. In this latter scenario, serine catabolism via serine hydroxymethyltransferase 2 (SHMT2) provides reducing equivalents to sustain NADPH production (which is critical for lipid synthesis and the preservation of redox homeostasis)^79,88^. Cytosolic malic enzyme 1 (ME1) mediates a similar function in pancreatic duct adenocarcinomas (PDACs) and highly proliferating breast cancers, ensuring the synthesis of NADPH from glutamate^72,89^. Interestingly, mitochondrial ME2 is deleted in a fraction of human PDACs, which renders them dependent on ME3-driven NADPH synthesis for survival and proliferation^90^.

Acetyl-CoA-derived acetoacetate also supports cancer proliferation by boosting BRAF kinase activity and consequently MAPK signaling^91,92^. Along similar lines, slightly elevated levels of ROS stimulate proliferation by inactivating tumor suppressors such as phosphatase and tensin homolog (PTEN) or by stabilizing HIF1A^93,94^. Moreover, physiological ROS levels contribute to the regulation of mitochondrial dynamics^95^, which is intimately involved not only in mitochondrial biogenesis, but also in the control of mitochondrial metabolism^96^. In line with this notion, multiple tumors overexpress ATPase inhibitory factor 1 (ATPIF1), which favors the dimerization of ETC complex V to limit ATP production and (as a side effect) increases ROS generation^97,98^. Intriguingly, ROS-driven cellular senescence can paradoxically support proliferation in a cell-extrinsic manner, as it sustains the secretion of mitogenic factors that act on neighboring cancer cells with intact proliferative capacities^99,100^. These observations exemplify the fundamental role of mitochondrial products at the interface of metabolism and signaling.

### Resistance to spontaneous RCD

Progressing neoplasms encounter harsh microenvironmental conditions (e.g., hypoxia, low nutrient availability, growth factor withdrawal), which would normally drive mitochondrial RCD via MOMP or MPT^32,33^. Malignant cells, however, acquire several alterations that increase the mitochondrial threshold for irreversible permeabilization, beyond the overexpression of BCL2 family members (see above)^101^. Some (but not all) tumors are characterized by an elevated mitochondrial transmembrane potential (Δψ_m_) linked to high glycolytic rates and increased resistance to RCD^102^. In this scenario, restoring pyruvate generation with chemical PDK1 inhibitors appears to be sufficient to cause RCD and inhibit tumor growth *in vivo*^102^. Similarly, detaching hexokinase 1 (HXK1) or HXK2 – the enzymes that convert glucose into glucose-6-phosphate in the first step of glycolysis – from mitochondria has been proposed to cause MOMP in cancer cells of different origin^103^. Moreover, the increased abundance of reduced glutathione that originates from a proficient reductive metabolism prevents cytochrome *c*, somatic (CYCS) from oxidation, which limits its capacity to activate apoptotic RCD upon MOMP^104^. The maintenance of optimal antioxidant defenses is also fundamental for cancer cells to avoid ROS-driven MPT, and oncogene signaling, glycolysis, as well as reductive glutamine carboxylation play a major role in this sense^88,105,106^. Interestingly, such a defense mechanism — which is partially related to the Warburg effect — appears to be conserved in yeast^107^. That said, slightly elevated ROS levels may increase the resistance of cancer cells to RCD by (1) triggering an adaptative hormetic response reminiscent of ischemic preconditioning^108,109^, and/or (2) promoting autophagy activation^110^. Interestingly, the supramolecular entity responsible for MPT, the so-called “permeability transition pore complex” operates in the context of physical and functional interactions with ETC components (notably, complex V) and other constituents of the molecular machinery for mitochondrial ATP synthesis^98^. In several cancer cells, proficient ATP production by mitochondria is associated with optimal Ca^2+^ homeostasis and limited MPT sensitivity^111^. Mitochondrial dynamics is also involved in the increased resistance of cancer cells to MOMP and MPT. Malignant cells cope with glucose deprivation by shifting to OXPHOS upon mitochondrial elongation secondary to dynamin 1-like (DNM1L) inhibition^112^, which is important to generate an efficient mitochondrial network upon the mitophagic removal of dysfunctional components^113^. Taken together, these observations suggest the existence of an intimate and bidirectional link between metabolism and mitochondrial RCD control.

### Diversification and interaction with the stroma

Progressing malignancies acquire a high degree of phenotypic and metabolic plasticity as they establish functional interactions with non-transformed components of the tumor microenvironment^114,115,116^. Both these aspects of the biology of malignant cells have been largely overlooked by studies based on cultured cancer cell lines. Recent *in vivo* work revealed that not only the oncogenic driver, but also the tumor microenvironment (in particular tissue of origin) influence the metabolic profile of malignant cells^117,118,119^.

One of the (hitherto debated) models of tumor evolution proposes the existence of a cancer stem cell (CSC) population endowed with self-renewing ability and responsible for both local progression and recurrence^120^. As compared to their more differentiated counterparts, CSCs from multiple malignancies including osteosarcoma, glioblastoma, and breast cancer display a predominantly glycolytic metabolism^121,122,123^. However, CSCs from other tumors such as ovarian cancer appear to primarily rely on OXPHOS for ATP synthesis^124^. Interestingly, different subsets of CSCs from the same tumor have been reported to preferentially catabolize glucose in a disparate manner^125,126^, suggesting that an additional layer of heterogeneity may exist within the CSC compartment to favor metabolic plasticity^127,128^. That said, the study of CSCs is complicated by the lack of widely accepted surface biomarkers for isolation, as well as by the tendency of these cells to rapidly evolve in culture. This implies that additional investigation is required to elucidate the precise metabolic profile of CSCs from different tumors and whether mitochondrial metabolism may offer targets for therapeutic interventions in this setting.

Prostate cancer cells reprogram tumor-associated fibroblasts (TAFs) toward anaerobic glycolysis, resulting in lactate secretion in the microenvironment and lactate-driven oxidative metabolism in malignant cells^129^. Along similar lines, PDAC cells drive TAFs into autophagic responses that ultimately sustain tumor growth by increasing the local availability of alanine (employed by cancer cells as a carbon source)^130^. Extracellular proteins can also be utilized by PDAC cells for carbon supply upon macropinocytosis^131^, but thus far no mechanisms whereby cancer cells stimulate protein secretion by non-transformed components of the tumor microenvironment for nutritional purposes have been described. Along similar lines, prostate, ovarian, breast, and colorectal cancer cells have been shown to obtain fatty acids for oxidative metabolism from local adipocytes, providing a support to tumor progression^132,133,134,135^. These observations exemplify parasitism-like relationships established by malignant cells in the tumor microenvironment. In addition, cancer cells can engage in metabolic competition for nutrients at limited availability, such as glucose and tryptophan, with immune effector cells (which reflects the metabolic similarities between highly proliferating cells)^136,137,138^. Such a competition is expected to influence the likelihood of natural immunosurveillance to control tumor progression. Finally, cancer cells from different regions of the tumor have been proposed to engage in a metabolic symbiosis involving the transfer of glycolysis-derived lactate from hypoxic to normoxic areas, where it would be employed to fuel OXPHOS (as a strategy to avoid competition for glucose)^139,140^. Additional investigation is required to elucidate the actual pathophysiological relevance of this process in human malignancies.

### Metastatic dissemination

The term metastatic dissemination (also known as metastatic cascade) generally refers to a multi-step process whereby cancer cells acquire the ability to colonize and form macroscopic lesions at distant sites^141^. Although macrometastases are generally considered as glycolytic entities (because they are often detectable by ^18^F-FDG PET), this is not always the case^142^. One of the first alterations of the metastatic cascade is the so-called epithelial-to-mesenchymal transition (EMT), which endows malignant cells with increased invasive potential^143^. Several mitochondrial metabolites favor the EMT^144^, in particular fumarate (owing to its ability to repress the transcription of the antimetastatic microRNAs upon inhibition of TET dioxygenases)^145^. Optimal mitochondrial biogenesis and OXPHOS seem also to be required for metastatic dissemination, as demonstrated upon silencing of the master regulator PPARG coactivator 1 alpha (PPARGC1A, best known as PGC-1α) in models of breast cancer^146^, and upon silencing of family with sequence similarity 210 member B (FAM210B) in models of ovarian cancer (resulting in PDK4 downregulation and consequent utilization of glycolytic pyruvate in the TCA cycle)^147^. Moreover, local invasion relies (at least in part) on oxidative mitochondrial metabolism at the cellular leading edge, resulting in cytoskeletal alterations required for motility^148,149,150^. Mitophagic defects also promote metastatic dissemination^151^, most likely by favoring mild ROS overproduction^152,153,154^. ROS indeed activate several signal transduction cascades associated with metastatic dissemination, including SRC and protein tyrosine kinase 2 beta (PTK2B) signaling^153,155^. In line with this notion, a genetic signature of mitochondrial dysfunction has been associated with metastatic dissemination and dismal prognosis in patients affected by nine different tumors^156^. Of note, imbalances in mitochondrial dynamics have also been linked with mild ROS overproduction and consequent metastatic dissemination^157,158^. Conversely, in the presence of severe oxidative stress, ROS *de facto* inhibit metastatic dissemination, most likely as a direct consequence of reduced fitness and RCD or cellular senescence^159,160,161^. In summary, although established macrometastases are generally characterized by elevated glucose uptake (presumably reflecting an intense glycolytic metabolism that boosts antioxidant defenses)^107^, OXPHOS and consequent ROS generation (provided that it remains below a cytotoxic threshold) are required for previous steps of the metastatic cascade. Most likely, there is a considerable heterogeneity in the extent to which metastatic lesions of different origin^117^ or at different anatomical locations^162^ actually rely on glycolytic versus respiratory metabolism. Further investigation is required to shed light on all the factors that influence the metabolic profile of macrometastatic lesions.

**Figure 2:**
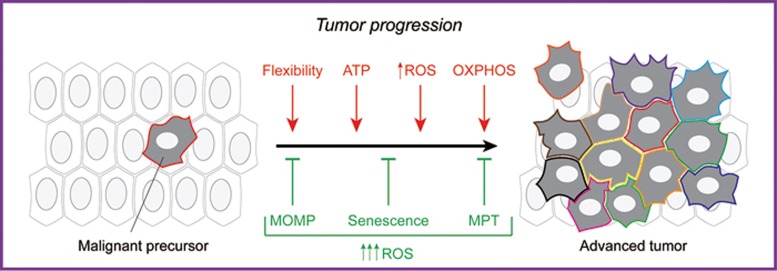
Mitochondrial metabolism in tumor progression. Mitochondria influence multiple processes that underpin tumor progression, including the proliferation of transformed cells, their resistance to adverse microenvironmental conditions, their diversification, their interaction with the tumor stroma and their dissemination toward distant anatomical sites. In particular, (1) mitochondria are major sources of ATP and building blocks for the proliferation of malignant cells; (2) progressing cancer cells display an increased threshold for mitochondrial outer membrane permeabilization (MOMP) and mitochondrial permeability transition (MPT), which renders them less sensitive to harsh microenvironmental conditions; (3) slightly supraphysiological levels of mitochondrial reactive oxygen species (ROS) foster tumor diversification (herein represented with assorted plasma membrane colors) by favoring mutagenesis; (4) different subsets of malignant cells exhibit differential metabolic profiles, which are important for their survival and function; (5) the metastatic cascade relies on optimal mitochondrial biogenesis and oxidative phosphorylation (OXPHOS), at least at the initial dissemination step. However, imbalanced ROS overproduction consequent to severe mitochondrial dysfunction is generally incompatible with tumor progression, resulting in MOMP- or MPT-driven regulated cell death or cellular senescence.

Altogether, these considerations suggest that mitochondria reside at a preferential hub connecting metabolism and signaling that is fundamental for tumor progression (Figure 2).

## Mitochondrial metabolism and therapeutic responses

The ultimate objective of conventional chemotherapeutics, targeted anticancer agents, radiation therapy as well as immunotherapy is to elicit the death or permanent inactivation (via cellular senescence or terminal differentiation) of malignant cells (directly and/or as a consequence of immunological mechanisms)^6^. Mitochondria are critically involved in the control of RCD triggered by all these treatments, implying that alterations of the propensity of mitochondria to undergo MOMP or MPT underlie a majority of cases of primary and acquired resistance^163,164,165,166^. As mentioned above, this notion drove an intensive wave of research aimed at the identification of molecules that would kill transformed cells or sensitize them to treatment by priming MOMP or MPT, such as the FDA-approved agent venetoclax^21^. Discussing the regulation of apoptotic and necrotic RCD by mitochondria in details goes beyond the scope of the present review^167,168^. That said, however, it should be noted that (1) RCD regulation at mitochondria involves a robust metabolic (rather than purely structural) component; (2) several metabolic aspects of the mitochondrial biology also influence therapeutic responses^101,169^ and (3) metabolic enzymes residing within mitochondria such as mutant IDH2 are being harnessed for the development of anticancer agents promoting terminal differentiation^45,170,171^.

BRAF^V600E^ inhibition with the FDA-approved agent vemurafenib is associated with a switch from glycolysis to OXPHOS, which is required for melanoma cells to resist treatment^172^. In this model, the ETC inhibitor honokiol is sufficient to abrogate resistance and restore cancer cell killing by vemurafenib^172^. Oncogene ablation in KRAS^G12D^-driven PDAC cells results in the selection of a subpopulation of cells predominantly relying on OXPHOS for energy production^173^. A similar switch from glycolysis to OXPHOS has also been documented upon MYC/KRAS or MYC/ERBB2 ablation in breast cancer cells^174^, and in the context of acquired resistance to phosphoinositide-3-kinase (PI3K) inhibition in glioma cells^175^. Moreover, resistance to PI3K inhibition in breast cancer cells has been linked to a switch from glucose to lactate as a main source of carbon units^176^. The activity of various transporters of the ATP-binding cassette (ABC) family – which support chemoresistance as they export a wide spectrum of xenobiotics – depends on OXPHOS-derived ATP availability^177^. In some cases, the expression of ABC transporters and the consequent acquisition of a chemoresistant phenotype stems from OXPHOS-driven inflammatory reactions culminating in the secretion of interleukin 6 (IL6) and tumor necrosis factor (TNF) into the tumor microenvironment^178^. Thus, in cells with a predominantly glycolytic metabolism, OXPHOS can promote resistance to treatment via both cancer cell-intrinsic and cell-extrinsic pathways. Conversely, malignant cells that predominantly utilize OXPHOS for energy production, including pancreatic CSCs, can become resistant to ETC inhibition as they acquire a partially glycolytic metabolism depending on MYC expression^179^. Likewise, chemoresistant ovarian cancer cells display a switch from OXPHOS to glycolysis accompanied by a PPP-dependent surge in antioxidant defenses^180^. Taken together, these observations suggest that the ability of most (if not all) cancer cells to flexibly rewire their mitochondrial metabolism underlies multiple instances of chemoresistance. This holds true for antineoplastic agents other than conventional chemotherapy, including radiation therapy^181^, antiangiogenic drugs^182,183,184^, and natural killer (NK)-based immunotherapy^185^. In this latter case, OXPHOS supports the resistance of cancer cells to NK cell-mediated lysis as it promotes the expression of MHC class I molecules (potentially resulting in restored sensitivity to CTL-mediated lysis)^185^.

**Figure 3:**
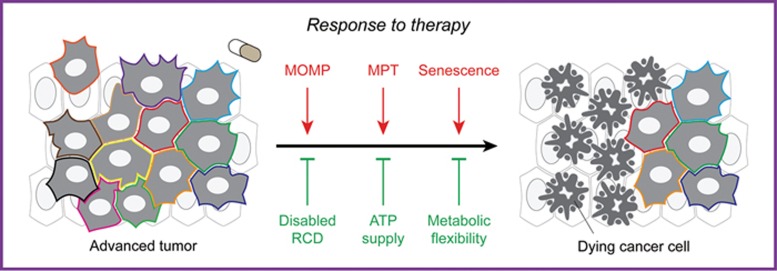
Mitochondrial metabolism in response to treatment. All forms of treatment, including chemotherapy, radiation therapy and immunotherapy, aim at triggering the demise — via regulated cell death (RCD) — or permanent inactivation — via cellular senescence — of malignant cells (directly, or as a consequence of immunological mechanisms). Thus, mitochondria control therapy-driven RCD in cancer cells, implying that alterations in the molecular mechanism underpinning mitochondrial outer membrane permeabilization (MOMP) and mitochondrial permeability transition (MPT) are a major source of resistance. Moreover, mitochondrial ATP fuels several pumps of the ATP-binding cassette family, hence fostering chemoresistance upon the extrusion of xenobiotics from malignant cells. Finally, the ability of malignant cells to flexibly switch between glycolysis and oxidative phosphorylation appears to play a major role in multiple instances of resistance to oncogene inhibition.

Thus, different forms of treatment establish compensatory metabolic networks that support cancer cell survival. Importantly, such metabolic perturbations may provide targets for the development of novel agents that sensitize cancer cells to treatment. Preclinical evidence in support of this notion is accumulating^186^. In summary, besides controlling multiple forms of RCD, mitochondria impact the response of cancer cells to treatment via metabolic rewiring (Figure 3).

## Mitochondrial metabolism in immunosurveillance

Mitochondria influence immunosurveillance via both cancer cell-intrinsic and cancer cell-extrinsic mechanisms. On the one hand, mitochondria are the source of many danger signals released by cancer cells as they die, and these signals are crucial for the activation of dendritic cells (DCs) to optimally prime tumor-targeting immune responses^187^. On the other hand, mitochondrial metabolism is involved in many functions linked to anticancer immunity, including (but not limited to) inflammasome activation, the establishment of protective immunological memory as well as the differentiation and tumoricidal activity of specific macrophage subsets^188,189^.

The best characterized mitochondrial product that participates in the elicitation of immune responses to dying cancer cells is ATP^190^. Extracellular ATP — which dying cancer cells can release in considerable amounts only if they can mount autophagic responses before death^191,192^ — mediates indeed prominent immunostimulatory and chemotactic functions upon binding to purinergic receptor P2X 7 (P2RX7) and purinergic receptor P2Y2 (P2RY2), respectively, on the surface of DCs or their precursors^193,194,195^. In line with this notion, autophagy-deficient malignant cells lose the ability of driving anticancer immunity as they succumb to chemotherapy or radiation therapy *in vivo*, a detrimental effect that can be partially corrected by inhibiting extracellular ATP degradation by ectonucleoside triphosphate diphosphohydrolase 1 (ENTPD1; best known as CD39)^191,196,197^. Moreover, autophagy activation with caloric restriction or molecules that mimic the biochemical effects of starvation boosts the therapeutic efficacy of immunogenic treatment modalities (including anthracycline-based chemotherapy) in rodent tumor models, an effect that is abolished by the depletion of ATG5 or ATG7 as well as by the overexpression of CD39^196,198,199^. Mitochondria contain many other molecules that can operate as extracellular danger signals, including (but not limited to) *N*-formylated peptides and mtDNA^187^. However, while the relevance of some of these molecules in other disease settings (e.g., systemic inflammatory response syndrome) is well-established^200^, their role in anticancer immunity remains to be fully elucidated. Indeed, the receptor for *N*-formylated peptides (which is expressed by DCs) appears to be required for dying cancer cells to elicit a tumor-targeting immune response, but it does so by binding to another danger signal, i.e., annexin A1 (ANXA1)^201^. That said, the release of mtDNA upon MOMP promotes the secretion of type I interferon by malignant cells, and this is required for the activation of optimal anticancer immune responses upon chemotherapy and radiation therapy^202,203,204,205^. Thus, mtDNA also operates as an intracellular danger signal to connect intracellular stress responses to the preservation of extracellular homeostasis^206^.

CTLs and helper T cells responding to antigenic stimulation engage in a proliferative response that — similar to cancer cell proliferation — extensively relies on glycolysis and is supported by mitochondrial fragmentation^207,208,209^. In addition, mitochondrial ROS are required not only for proximal TCR signaling, but also for the activation of multiple transcription factors necessary for optimal T-cell functions, such as NF-κB and nuclear factor of activated T-cells 1 (NFATC1; best known as NFAT)^210,211^. At odds with their effector counterparts, memory T cells predominantly rely on fatty acid oxidation and OXPHOS to support their metabolic needs, a result of a metabolic reprogramming that involves not only mitochondrial elongation but also mechanistic target of rapamycin complex 1 (MTORC1) inhibition coupled to autophagy activation^208,212,213^. Intriguingly, a similar metabolic profile is also displayed by immunosuppressive cell types including CD4^+^CD25^+^FOXP3^+^ regulatory T cells and myeloid-derived suppressor cells^214,215^, which presumably renders them less sensitive to metabolic competition for glucose within the tumor microenvironment.

Macrophage polarization and activity are also influenced by mitochondrial metabolism. On the one hand, inhibition of the ETC appears to promote the differentiation of macrophages toward a pro-inflammatory and tumoricidal state (generally referred to as M1), which display a predominantly glycolytic metabolism secondary to the autophagic removal of mitochondria^216,217,218^. Conversely, M2-polarized macrophages, which generally exert tumor-supporting functions, preferentially employ OXPHOS as a source of ATP, especially in hypoxic conditions^219,220^. However, the oxidative burst that underlies the phagocytic activity of M1 macrophages depends on ROS of direct or indirect (via NADPH) mitochondrial derivation^221^. A similar consideration applies to the pro-inflammatory activity of M1 macrophages, which relies on ROS-dependent NF-κB transcriptional responses as well as on the activation of the so-called inflammasome, a supramolecular platform that produces IL1β and IL18 in a ROS- and mtDNA-dependent manner^222,223^.

**Figure 4:**
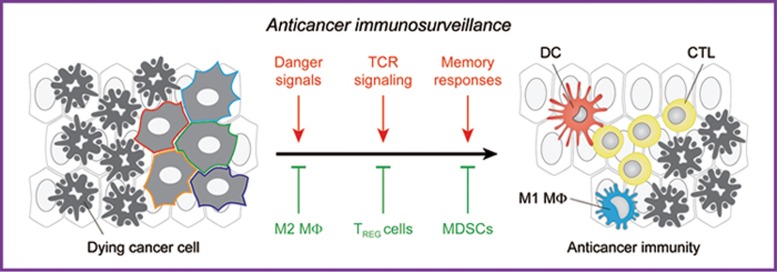
Mitochondrial metabolism in immunosurveillance. Mitochondria are fundamental for the recognition of cancer cells by the immune system, as well as for the consequent activation of a tumor-targeting immune response. On the one hand, mitochondrial products including ATP, reactive oxygen species (ROS) and mitochondrial DNA (mtDNA) operate as danger signals, either extracellularly (like ATP) or intracellularly (like ROS and mtDNA). On the other hand, mitochondrial ROS are required for T-cell activation in response to TCR engagement, and oxidative phosphorylation (OXPHOS) is required for the establishment of immunological memory as well as for the tumoricidal and pro-inflammatory activity of M1 macrophages (MΦ). However, OXPHOS also supports the differentiation of immunosuppressive cells including M2 macrophages, CD4^+^CD25^+^FOXP3^+^ regulatory T (T_REG_) cells and myeloid-derived suppressor cells (MDSCs). CTL, cytotoxic T lymphocyte.

Taken together, these observations exemplify the intricate involvement of mitochondrial metabolism in anticancer immunosurveillance (Figure 4).

## Concluding remarks and perspectives

**Figure 5:**
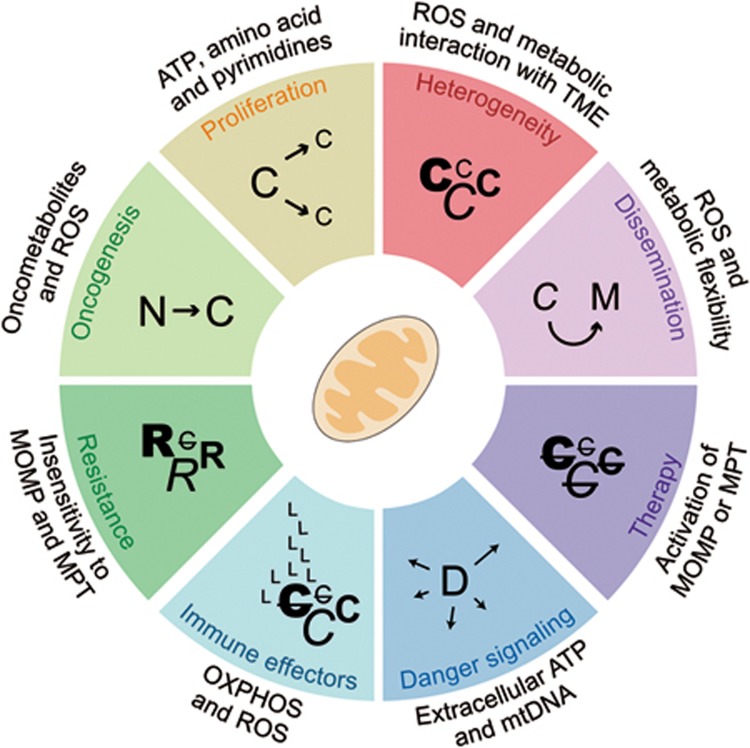
Mitochondrial metabolism and oncogenesis. Mitochondria have a major impact on virtually all processes linked to oncogenesis, encompassing malignant transformation, tumor progression, response to treatment and anticancer immunosurveillance. C, cancer cell; D, dying cancer cell; L, lymphocyte; M, metastatic cancer cell; mtDNA, mitochondrial DNA; MOMP, mitochondrial outer membrane permeabilization; MPT, mitochondrial permeability transition; N, normal cell; OXPHOS, oxidative phosphorylation; R, resistant cancer cell; ROS, reactive oxygen species; TME, tumor microenvironment.

Mitochondria have attracted considerable attention as targets for the development of novel anticancer agents, not only because they have a central role in the resistance of malignant cells to RCD induction by treatment, but also because they underlie their phenotypic and metabolic plasticity (Figure 5). The case of venetoclax, a molecule that triggers RCD by mimicking the activity of pro-apoptotic members of the BCL2 protein family, well exemplifies the high potential of agents targeting mitochondria for the treatment of specific malignancies^21^. However, non-specifically targeting mitochondrial functions within the tumor microenvironment may have major unwarranted effects including the inhibition of anticancer immune responses, a situation that reminisces the use of pharmacological inhibitors of autophagy^224^. Thus, refined strategies that allow for specifically modulating mitochondrial functions in selected cell populations will have to be devised for the therapeutic potential of mitochondria-targeting agents to be fully harnessed in the clinics. A large body of preclinical and clinical work is still required for this ambitious objective to become a clinical reality.

## Competing Financial Interests

NF is a consultant to Lyric Pharmaceuticals (South San Francisco, CA, USA). LG provides remunerated consulting to OmniSEQ (Buffalo, NY, USA). The remaining authors declare that they have no competing interests.

## Abbreviations

^18^F-FDG: (2-[^18^F]fluoro-2-deoxy-𝒟-glucose)
2-HG: (2-hydroxyglutarate)
α-KG: (α-ketoglutarate)
Δψ_m_: (mitochondrial transmembrane potential)
CSC: (cancer stem cell)
CTL: (cytotoxic T lymphocyte)
DC: (dendritic cell)
EMT: (epithelial-to-mesenchymal transition)
ETC: (electron transport chain)
FA: (Fanconi anemia)
MOMP: (mitochondrial outer membrane permeabilization)
MPT: (mitochondrial permeability transition)
mtDNA: (mitochondrial DNA)
NK: (natural killer)
OXPHOS: (oxidative phosphorylation)
PDAC: (pancreatic duct adenocarcinoma)
PET: (positron emission tomography)
PPP: (pentose phosphate pathway)
RCD: (regulated cell death)
ROS: (reactive oxygen species)
TAF: (tumor-associated fibroblast)
TCA: (tricarboxylic acid)
